# Quantitative Variations of Intracellular Microcystin-LR, -RR and -YR in Samples Collected from Four Locations in Hartbeespoort Dam in North West Province (South Africa) During the 2010/2011 Summer Season

**DOI:** 10.3390/ijerph9103484

**Published:** 2012-10-02

**Authors:** Elbert A. Mbukwa, Titus A.M. Msagati, Bhekie B. Mamba

**Affiliations:** Department of Applied Chemistry, Faculty of Science, University of Johannesburg, P.O. Box 17011, Doornfontein 2028, Johannesburg, Republic of South Africa; Email: mbukwaelbert@gmail.com (E.A.M.), bmamba@uj.ac.za (B.B.M.)

**Keywords:** summer, Hartbeespoort Dam, dry weight (DW), quantitative LC-ESI-MS, intracellular variations, microcystin-RR

## Abstract

The Hartbeespoort (HBP) Dam is a reservoir used for agricultural, domestic supply of raw potable water and recreational activities in South Africa’s North-West Province. Eutrophication and cyanobacterial blooms have long been a cause of water-quality problems in this reservoir. The most prevalent bloom-forming species is *Microcystis aeruginosa*, often producing the toxin microcystin, a hepatotoxin which can negatively impact aquatic animal and human health, and poses a problem for potable water supply. Algal samples were collected monthly from four pre-determined sites in the dam during the summer months (December 2010–March 2011). Intracellular microcystins (MCs) were extracted using SPE C_18 _cartridges, followed by separation, identification and quantification using LC-ESI-MS techniques. Quantitative variation studies of MCs were conducted with respect to MC congener isolated, sampling site and month. Three main MC congeners (MC-RR, -LR and-YR) were isolated, identified and quantified. In addition, three minor MCs (MC-WR, MC-(H_4_)YR and (D-Asp^3^, Dha^7^)MC-RR were also identified, but were not quantified. The MC dominance followed the order MC-RR>MC-LR>MC-YR across all sites and time. The maximum and minimum concentrations were 268 µg/g and 0.14 µg/g DW for MC-RR and MC-YR, respectively, of the total MCs quantified from this study. One-way ANOVA showed that there were no significant differences between average MC concentrations recorded across months (*P* = 0.62), there was, however, a marginally-significant difference in concentrations among MC congeners (*P* = 0.06). ANCOVA revealed a highly significant interaction between sites and MC congeners on MC concentration (*P* < 0.001).

## 1. Introduction

**Figure 1:**
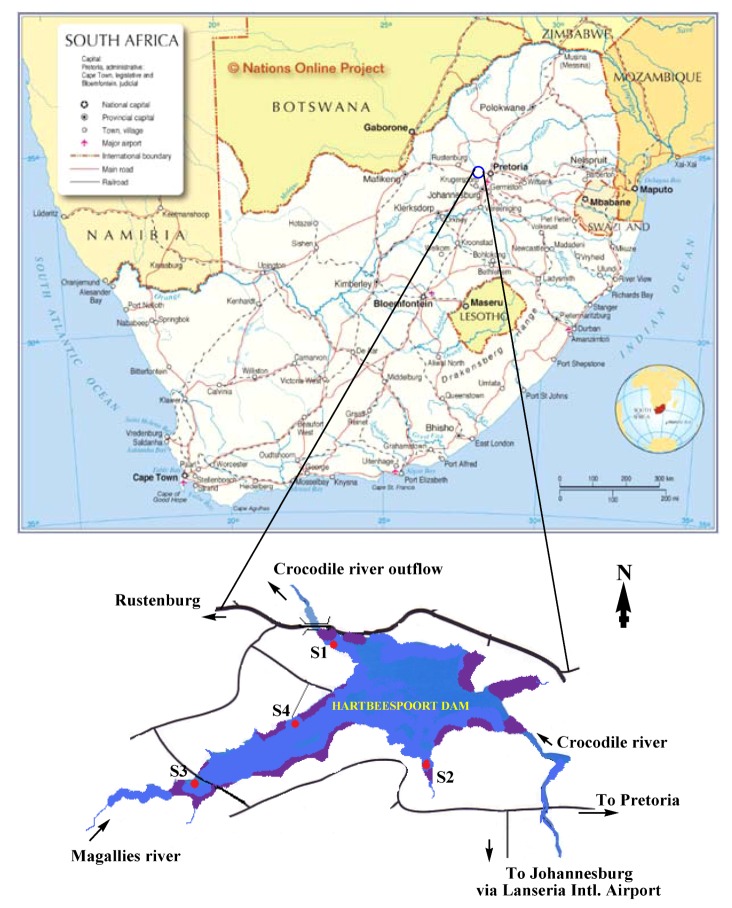
Map and GPS co-ordinates showing sampling sites on the Hartbeespoort (HBP) dam. S1: (−25°43'38.76"S, +27°50'57.21"E), S2: (−25°46'14.09"S, +27°51'57.85"), S3: (−25°45'37.71"S, +27°48'5.72"E) and S4: (−25°44'56.87"S, +27°50'0.05"E). Conservation/or restricted areas in the dam are marked with purple colour (source: African & South African maps: Nations Online Project available at www.nationsonline.org/oneworld/map/south_africa_map.htm).

Hartbeespoort Dam is situated in the North-West Province of South Africa. The reservoir is fed by the waters of the Crocodile and Magalies Rivers (Figure 1) and has a mean depth of 9.6 m, maximum depth of 45.1 m and surface area of 20 km^2^. Hartbeespoort Dam is renowned for its poor water quality and is arguably one of the World’s worst examples of eutrophication, due to the high nutrient loads which enter the system and have overburdened the reservoir basin for decades. 

Microcystins (MCs), a group of cyclic heptapeptide hepatotoxins are produced by a number of cyanobacterial genera [1,2]. Freshwater microcystin-producing cyanobacterial species, including *Microcystis* spp., *Anabaena *spp., *Planktothrix *(*Oscillatoria*) spp., *Aphanizomenon *spp. and* Nostoc *spp. [1,2], are on the increase worldwide due to increased environmental and water pollution leading to eutrophication of aquatic environments [1,2,3,4,5]. Microcystins are characterised by the presence of a unique non-proteinogenic β-amino acid called ADDA [6,7,8,9]. The toxicity of MCs is highly dependent on the ADDA group, the MeDha group (MeDha *= N*-methyldehydroalanine) and the structural cyclic nature [10,11]. Over 80 known microcystin structures (variants) have been reported worldwide from freshwater systems with their variations being mainly due to amino-acid replacements either at position 2 or 4 of the MC backbone [12], for details on the general structure refer to Sivonen *et al.* [8]. Other structural variations have been attributed to methylation/or demethylation processes of the methyl groups on amino acids at positions 3 and/or 7 [7,8]. The ADDA group [6] is highly conserved in microcystins (and nodularins) and stable against physiological replacements by other amino acids. Due to this stability, the ADDA group has therefore been utilised in various methods for the identification of microcystins (and nodularins) as described in Msagati *et al.* [13], these include ELISA [14], HPLC-UV/PDA [15,16,17,18,19], LC-*ESI*-MS [18,19], *etc.* In positive mode LC-*ESI*-MS the ADDA group gives a characteristic fragment ion at *m/z* 135 corresponding to the [phenyl-CH_2_CH(OCH_3_)]^+^ ion [13]. This ion therefore is often used as a diagnostic feature for the identification of MCs in algal samples [13,18] when using the LC-*ESI*-MS technique. The LC-MS technique gives reliable data with regard to detection, identification, quantification, differentiation and discovery of new MC congeners than other methods stated above [20]. For instance, it has been observed that ELISA is prone to cross-reactivity with MCs other than the MC-LR variant and thus its use is limited whenever identification and quantification of specific MC variants in samples is necessary [21,22,23,24,25]. However, the LC-MS offers complementary results for accurate detection and quantification of the same [7,18].

Cyanobacterial cell deaths caused by ageing, stress, mechanical breakdown or chemical activities (e.g., grazing, water purification processes, pump suctions, boat propellers, *etc.*) result in releases of intracellular MCs and increased extracellular MCs into the surrounding water [26,27,28,29,30]. Extracellular MCs are both highly soluble and relatively stable in water [31] posing a worldwide health concern. Microcystin toxicities resulting in human illnesses, as well as wildlife and fish kills, have been reported [2,3,10,17,32,33,34,35,36,37], including many cases in South Africa [38]. The characteristic solubility of MCs in water therefore makes these toxins bioavailable for a couple of days in a water column [23,31] before they can be completely biodegraded. The accumulation of MCs in marine organisms [39], aquatic plants and other life forms found in water environments [23,40,41,42] has been reported. 

Microcystin loading and spatial distribution in a given aquatic ecosystem is a function of intracellular MC content in algal cells and is closely related to algal species dynamics and dominance [43]. Papadimitriou *et al.* [40] showed that extracellular MC concentrations extracted from water samples were lower than intracellular MC concentrations extracted from algal cells collected from the same location and time, indicating dangers of possible underestimation or overestimation of MC concentrations should quantification be based solely on extracellular MCs. Lower estimates in extracellular MC levels compared to total intracellular MC levels are due to a number of factors, including algae grazers , assimilation of bioavailable MCs by aquatic organisms, wash-away by water drifts, adsorption on suspended solids/organic matter/sediments, MC degradations [44], *etc.* Thus, depending on the method used, measuring intracellular MC concentrations gives more complete information on the quantity and type of MCs, species distribution and their dynamics rather than using extracellular MC concentrations [45].Seasonal and temporal variations in cyanobacteria population, particularly with *M. aeruginosa* dominance in Hartbeespoort Dam, have been studied [24,38,46,47,48] and shown to be related to MC production as described elsewhere [29,43,49]. However, to the best of our knowledge and based on the literature surveyed, little information exists in terms of quantitative assessment of intracellular MC with respect to congener concentration variations, dominance and distribution in the Hartbeespoort Dam. In terms of quantitative studies on MCs commissioned and funded by the Water Research Commission (WRC), South Africa (www.wrc.org.za), including some recent works, the results presented were mainly based on total microcystin concentrations expressed as MC-LR equivalence using immunoassay or biochemical methods [25,50]. However, Conti *et al.* [51] demonstrated that besides immunoassay/biochemical methods being very sensitive tools for MC detection and quantification, their use results in over-estimations of toxins, probably due to cross-reactivity, false positives or false negatives [22,40,52,53,54,55]. Therefore, the objectives of the present study were to:

- Extract and identify intracellular microcystin congeners from algal blooms collected from four sites on the dam accessible by the public either directly or indirectly (by boat).
- Quantify intracellular microcystin congeners using the LC-*ESI*-MS technique and determine their spatial distribution with respect to locations (sites), time and MC type (congener).
- Establish an MC congener dominance profile for water-quality assessment with respect to the use of Hartbeespoort Dam water resources.

## 2. Materials and Methods

### 2.1. Sample Collection

Samples were collected monthly from December 2010 to March 2011 from four pre-determined sites in the Hartbeespoort Dam (Figure 1), based on four factors:

- Areas that can easily be reached either by boat or by walking along the banks of the dam (S1–S4).
- Relative distance to fishing/conservation area hotspots (S2–S4).
- Proximity to Magalies River inflow and Crocodile River outflow to/from the dam (S3 and S1, respectively).
- Proximity to the animal conservation area (zoo) and water purification station (S1).

Algal cells were collected in 500 mL glass bottles from each site during the summer season. Samples were transported to the laboratory in cooler boxes filled with ice blocks and processed the same day by filtration. For algal identifications, samples were treated according to [56,57] and analysed within 24 h of collection. 

### 2.2. Sample Pre-Treatment for Microcystin Extraction

Algal cells were filtered on pre-weighed GF/C glass-fibre filters (47 mm, 0.45 µM). Filters were washed further by flushing with distilled water to remove any superficial microcystins remaining on cells. Filters were stored at −20 °C before freeze-drying. After freeze-drying, weighed algal cells (1.0 g) were extracted using 70% MeOH_(aq) _and then the extracts were subjected to CHCl_3_/MeOH/H_2_O liquid-partitioning (7/6/3, v/v/v) to remove pigments, co-eluting compounds and other cartridge-blocking material [58] prior to SPE extraction using Waters^TM^ HLB cartridges [59].

### 2.3. Physicochemical Parameters and Species Identification

On-site physicochemical parameters (conductivity, temperature, pH and dissolved oxygen) were measured immediately at each site using multifunction meter (YSI^TM ^6-Series Sonde, Marion, Germany). The determination of the concentrations of chlorophyll-*a*, nitrates and phosphates as well as species identification were carried out in a parallel project that dealt with the assessment of nutrient loading in the Hartbeespoort Dam (Zoology Department, University of Johannesburg).

### 2.4. Analytical Standards, Reagents and Laboratory Materials

Organic solvents (MeOH, CHCl_3_, MeCN, HCOOH) were of high purity analytical grade and/or HPLC grade, >99% purchased from Merck (Johannesburg, South Africa) and/or Alfa Aesar (Karlsruhe, Germany). Microcystin standards (MC-RR, -YR and -LR) were purchased from CyanoBiotech GmbH (Berlin, Germany) and were supplied by Industrial Analytical (Pty) Ltd (Johannesburg, South Africa). SPE cartridges (Waters Oasis^TM^ HLB cartridge: 60 g, 3 mL) were purchased from Waters Inc. (Milford, MA, USA), and supplied by Microsep (Pty) Ltd, SA (Johannesburg, South Africa). Samples were filtered through Whatman GF/C glass-fibre filters (porosity 0.45 µM, Whatman International Ltd., Maidstone, UK). 

### 2.5. LC-ESI-MS Instrumentation and Conditions

The SPE extracts were analysed using a modified method reported in the literature [12]. Briefly, a Waters^TM^ LC-MS instrument (with a Waters^TM^ 3100 Mass Detector) coupled with an electrospray ionisation source (ESI) was conditioned for 10 min with the mobile phase shown below. MCs separation was performed on a conditioned C_18_ column (Waters Symmetry300^TM^, 4.6 mm × 75 mm, 3.5 μm) using the Alliance Waters^TM^ e2695 separation module. The mobile phase consisted of a mixture of 0.5% (FA) in Milli-Q water (A) and 100% acetonitrile (B). The column was operated at room temperature, and the microcystins were eluted within a gradient window of 20 min using a reverse phase system consisting of 25% B (10 min), 70% B (10 min), 25% B (11.1 min), 25% B (20 min). Flow rate and sample injection volumes were set at 0.5 mL/min and 0.5 μL, respectively. The ion source was operated on both positive and negative ESI modes for all experiments. Structural identification of microcystins in samples was based on retention time and fragment ion products relative to respective elution window/fragmentation pattern of authentic analytical standards, results were complemented with literature values. 

### 2.6. Microcystin Analysis and Quantification

Reference standard curves were established from linear regression values of respective authentic microcystin standards using Sigmaplot^TM^ Software (Version 8). Linear regression equations were established giving acceptable *R*^2^ values for each authentic standard solution. Major intracellular microcystins (MC-RR, -YR, and -LR) isolated from *M. aeruginosa* cells were quantified based on linear regression equations using peak areas [60,61,62]. All experiments on field samples were performed in triplicate and quantities were reported in mean values (*n *= 3). Statistical analyses (one-way ANOVA and ANCOVA) were performed on an R2.14.1 statistical package [63] using mean values of the calculated MC concentrations (*P *< 0.05 denoted a significant difference). 

## 3. Results and Discussion

### 3.1. Site Selection

The Hartbeespoort Dam (GPS co-ordinates: 25°43'44.56"S, 27°51'30.35"E) is one of the major water impoundments situated in North-West Province, South Africa, with its water being used for irrigation, domestic and recreation purposes [64]. Thus monitoring of the quality of its water is a primary priority for public health. However, on a daily basis, this reservoir receives millions of litres of treated wastewater that is rich in phosphates and nitrogenous species, this has led to excessive nutrient loading resulting in Hartbeespoort Dams being one of the most heavily eutrophied dams in South Africa [65,66]. Eutrophication of this dam dates back to beyond the 1970s and the water is quite often characterised by foul smell and heavy green pigmentation especially during summer seasons [67]. However, substantial lake restoration control measures are currently underway to reduce nutrient loading (phosphorus/nitrogen), and a shoreline restoration project is being undertaken [66]. Physical removal of algae and water hyacinth after trapping them with floating boom barriers is also an ongoing dam-remediation project managed by DWAF [68]. Sample-collection sites were pre-selected in areas where operational dam restoration and sampling activities would not have any effect/impact on each other. However, sample-collection sites were meant to give a large representative sample area of the dam with different features of interest relevant to public health including recreational and fishing activities and unrestricted/ease of public access (Sites S1–S4) (Figure 1). A pre-survey (and during GPS positioning exercise) showed that Site S1 experienced visible algal cell accumulation during the summer, probably due to the wind direction and effects of currents caused by the outflow of water near the water gates. In addition, this site (S1) was situated within a few metres from the water-purification facility for domestic use as well as to the animal conservation area (known as the Snake Park). Site S2 was selected due to low current and turbulence effects that led to massive algal scum throughout sampling. Algal blooms characteristically occur in calm, nutrient-rich water bodies [69]. Likewise, Site S3 was a relatively calm site, and protected from strong winds by some bushes forming part of a conservation scheme around the dam. The water mass around Site S3 is mainly from Magalies River inflow (Figure 1). Persistent small quantities of algal cells are very common around Site S3, even during the winter season (personal communication from a local resident). 

### 3.2. Microcystis spp. Identification

Preserved cells were identified microscopically according to the Utermöhl method as described [70]. *Microcystis aeruginosa* species was the dominant species throughout the sampling period across all sites with an average dominance rating of over 80% (*i.e.*, 80% to 100% of total cells identified belonged to *M. aeruginosa*), data not shown. These findings corroborate those reported in other previously published reports which state that over the past two decades *M. aeruginosa* has been dominant in Hartbeespoort Dam, particularly during the summer season [25,48,50,71,72]. Other algal species identified included *M. wesenbergii *(<10%), *Spirulina* (<5%), *Planktothrix* spp. (<5%), *Melosira* spp. (<1%) and *Nitzschia *spp. (<1%). However, although their presence in this dam has been previously reported [25,50,71], none of these minor species have been associated with MC production in Hartbeespoort Dam. Hence it is widely accepted that *M. aeruginosa* is the major producer of MCs observed in this dam. 

### 3.3. On-Site Environmental Conditions

Some important physicochemical parameters that prevailed in the dam during the summer are shown in Table 1. The proliferation and persistence of *M. aeruginosa* as well as MC production are dependent on a number of environmental conditions prevailing in any given water body [2,46,73,74,75]. While an increased N:P nutrient ratio is known to be crucial in this regard [76], other important parameters have previously been demonstrated to play an important role in MC production, including as demonstrated recently, optimal pH and temperature [75], as well as light intensity [25,75]. During the entire period of our study, the average pH levels in the dam ranged between pH 8.8 ± pH 0.21 to pH 7.4 ± pH 0.07. This pH range is suitable for desolvation of phosphates in a water system as demonstrated by Greenwald [77]. The availability of soluble phosphates in water increases as the pH of the solution increases towards alkaline conditions up to a pH < 8.6 [77]. The optimal pH range observed in Hartbeespoort Dam is a characteristic feature of most eutrophic waters [71,78] as it is suitable for metabolic activity and growth of toxic algae [5,71,75,79] at the expense of less favoured algae. Similar to previously reported pH values [25,38,71], floating algal cells were conspicuous on the water surface throughout the dam, including all sampling sites where *M. aeruginosa* cells were collected (Figure 1). The presence of larger masses of algal cells in some areas (e.g., Site S1 and Site S2), which were later shown to be dominated by *M. aeruginosa*, indicated that the dam conditions were favourable for *M. aeruginosa* growth, distribution and dominance as shown earlier [46,50]*.*

Algal growths were observable throughout sampling dates, during which the average temperature in December 2010 through to March 2011 ranged between 24.2 °C and 26.2 °C (Table 1). The observed temperature range is typical for summer seasons as reported elsewhere favouring the growth of *Microcystis* spp. [4,28,29,33,73,74,80] as is the case in Hartbeespoort Dam [25,50]. There was an increase in the quantities (>5.94 µg/g DW) of all MCs extracted towards mid-summer, particularly by February 2011 during which period the water surface in the dam was completely green, evidenced by higher quantities of chlorophyll-*a* measured (data not shown). Algal cell multiplications result in an increased algal biomass paralleled by an increased quantity of chlorophyll-*a* being produced, as well as elevated MC production [76,81]. Higher quantities of MCs were recorded at Site S2 (Figure 1-Figure 3) where chlorophyll-*a* concentration was the highest, being higher than S1, S3 and S4 (data not shown). A correlation between higher total MC concentrations expressed as MC-LR eq/L and higher chlorophyll-*a* concentrations have been reported for algal cells collected from this dam [25,47,50]. Areas around Site S1 and Site S2 have been identified as some of the historical algae concentration zones due to their locations and conditions that favour massive algal cell aggregations and growth [82].

**Figure 2:**
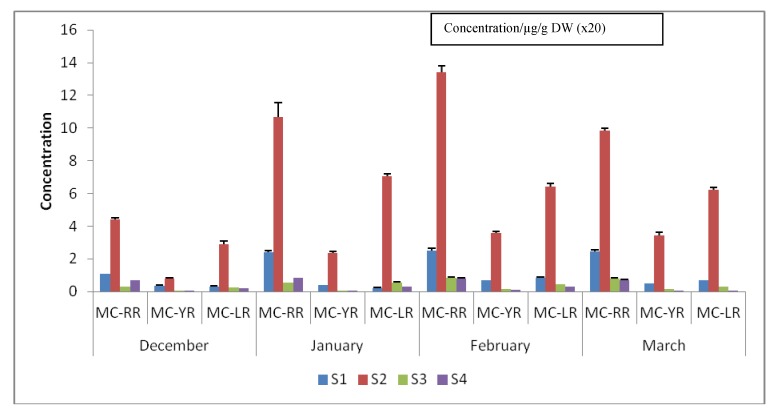
Graphical presentation of the quantitative variations of MC-RR, -LR and -YR among sites (S1 to S4) and months (December–March).

A decrease in DO is commonly associated with microbial activity taking place [75]. Towards the end of the summer season there was a tremendous decrease in DO across all sites, an indication that there was higher microbial activity, probably due to bacteria feeding on dying algal cells. A decrease in intracellular MCs extracted from all sites (S1 to S4) in March 2011 supports this observation (compare Figure 2 and Table 2). A decrease in intracellular MC towards the end of the summer is of particular importance in terms of public health since higher releases of extracellular MCs are expected due to cell deaths. Decreasing numbers of visible algal cells in a water column and clear water can be a deceiving water-safety indicator to users, should there be no post-bloom monitoring strategies to determine MCs in recreational water as well as in domestic water, particularly after the summer.

### 3.4. Isolation, Separation and Identification of MCs using LC-ESI-MS

Pooled MC-rich hydroalcoholic extracts from a partitioning step were dried under vacuum at 40 °C, then re-dissolved in 1 mL MeOH and subjected to the SPE protocol [16]. After LC-MS instrument and column conditioning (10 min), retention times (RTs) for pure and mixed standards were established and optimised. Similarly, SPE products of algal samples were injected into the column using the auto-sampler and eluted for 20 min as had been done for the standards. Three major microcystins, namely MC-RR, -YR, -LR and a few minor microcystin variants, namely MC-WR, MC-(H_4_)YR and (D-Asp^3^, Dha^7^)MC-RR were separated and identified based on m/z signals in positive *ESI* mode. The negative mode did not give good results, so it was not used. 

The observed molecular ion masses, fragments and retention times were analysed relative to those of the authentic standards used in this study and compared to the literature values. The assignments were as follows: MC-RR: (*m/z* 1,039.0 [M+H]^+^, ADDA *m/z* 135, RT 6.57), MC-YR: (*m/z* 1,046.3 [M+H]^+^, 523.3 [M+2H]^2+^, ADDA *m/z* 135, RT 7.54), MC-LR: (*m/z* 996.3 [M+H]^+^, ADDA *m/z* 135, RT 7.75) and MC-WR: (*m/z* 1,069.3 [M+H]^+^, ADDA *m/z* 135, RT 8.37). The ADDA fragment ion [PhCH_2_CH (OCH_3_)]^+ ^(*m/z* 135) resulted from α-cleavage of the methoxy group of the ADDA residue from microcystins [83,84] and was used as a diagnostic fragment for MC identification [13,18,85]. Except for MC-LA (not isolated in this study), our results are therefore in agreement with those of previous studies that reported on the occurrence of *M. aeruginosa* in Hartbeespoort Dam and the presence of various microcystin congeners including MC-RR, -YR, -LR, -LA and -WR [46,48,86]. From our work, further analysis of LC-*ESI*-MS data showed two other minor MCs from all algal extracts collected from all sites (December–March), which is also an indication that the species producing these MCs dominates during summer and is well distributed in the whole dam. Structures of the minor MCs corresponded to MC-(H_4_)YR (*m/z* 1050, [M +H]^+^, *m/z* 135, ADDA) [83] and (D-Asp^3^, Dha^7^) MC-RR (*m/z* 1010, [M+H]^+^, *m/z* 135, ADDA) when compared with information gleaned from the literature [87]. These two MCs (*i.e.*, MC-(H_4_)YR and (D-Asp^3^, Dha^7^)MC-RR) have previously been isolated from *M. aeruginosa* elsewhere [62,86], but there is limited information on their production from the same species found in the Hartbeespoort Dam, thus more investigation is underway to further elucidate their occurrences and to quantify them.

### 3.5. Quantification and Quantitative Variations of Intracellular MC-LR, -RR and -YR

**Figure 3:**
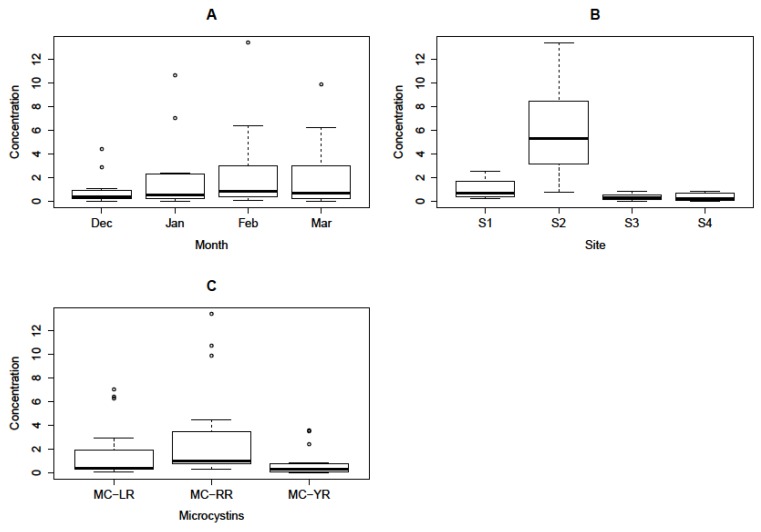
One-way ANOVA presentation of the statistical differences of mean MC concentrations (µg/g DWx20) for a study conducted between December and March. (**A**) Across months: No significant differences (*P *= 0.62). (**B**) Across sites: High significant difference (*P *< 0.001). (**C**) Among MC congeners: Marginal significant difference (*P *= 0.060).

*Microcystis aeruginosa* cells were identified from all samples collected from December 2010 to March 2011. Freeze-dried algal samples were treated similarly during MC extraction. To minimise instrumental and operational errors quantifications were performed in triplicate to generate mean values for each MC congener (Table 2). Complete removal of pigments and/or organic matter was achieved through liquid-partitioning procedures using CHCl_3_ as a non-polar solvent mixed with aqueous methanol prior to SPE [58]. When not properly removed from algal samples, green pigments and organic matter lead to SPE cartridge blockages, prolonged extraction time, and poor LC-MS spectra resulting in problematic quantification [88] and low yields of MCs due to pigment influenced degradation of MCs [89] and signal interferences [59]. Higher recoveries of MC-RR, -LR and -YR have been demonstrated following a chloroform-methanol partitioning step prior to SPE protocol [59] resulting in clean and quantifiable LC-MS spectra. The insolubility of MCs in chloroform is therefore of particular advantage in the isolation and LC-MS quantification of MCs following a partitioning step using chloroform-methanol solvent system prior to SPE protocol. In this study clean LC-MS spectra were obtained following a liquid-partitioning-SPE procedure (data not shown). Microcystin-RR, -LR and -YR were quantified using peak areas substituted on linear regression equations: Y = 5,342x − 4,218: (*R*^2 ^= 0.9963), Y = 4,030x − 2,518: (*R*^2^ = 0.9946), and Y = 3,098x + 1,172: (*R*^2^ = 0.9948) of standard curves for MC-RR, -LR and -YR solutions (0.5, 1.0, 2.0, 4.0, 6.0, 8.0 and 10.0 µg/mL), respectively. Monthly mean concentrations (*n *= 3) were calculated for each MC congener and total MC concentrations were shown (Table 2). Aquatic environments are dynamically non-uniform in nature leading to the existence of micro-environments within any given water system. The existence of micro-environments has been shown to influence and govern factors involved in the development of algal blooms and distributions, MC productions and quantitative variations, both in time and space [79]. Micro-environmental dynamics regulate important growth factors, particularly N/P nutrient ratios, thereby affecting growth rates of MC-producing algal blooms leading to variations in MC content [90,91]. From information given in Table 1 and Figure 2 it was shown that there were visible variations in the amounts of intracellular MCs among sites as well as on a monthly basis indicating that MC production was governed by differences in micro-environments within the dam [79]. Basically, MC-RR accounted for the highest amounts of all MCs quantified at all sites and over the four months of the sampling period (Table 1, Figure 2), however, statistically, a one-way ANOVA showed that there was no significant difference in MC concentrations among months, and however, marginal significant difference among MC congeners was observed (*P *= 0.62 and *P *= 0.06, respectively) (Figure 3A and C). On the other hand, a one-way ANOVA showed that there was a significant difference (*P *< 0.001) in MC concentrations among sites. The highest total MC concentration of about 468 µg/g DW was recorded at Site S2 in February 2011 (Table 2). These results support the information about historical massive concentrations of algae [82] around this area, particularly *M. aeruginosa* cells. A multivariate analysis to determine effects of interactions between sites and MC congener on MC concentration was performed using ANCOVA (Table 3). Results showed that there was a highly significant influence of the sampling sites (*P *< 0.001) resulting in quantitative inter-site (spatial) MC variations (Table 2 and Table 3). However, we did not find any significant interaction between period of sampling and site (*P *= 0.5764), a probable indication that the same species producing MCs was dominant throughout study. Microcystin content variations, including short-term (temporal), inter-site (spatial), perennial as well as seasonal variations, are a common trend in many eutrophic systems due to changing environmental factors and species succession/or dynamics [79]. Publications on intracellular and/or extracellular MC content variations, including temporal/short-term/or inter-site (spatial) variations, have appeared in which either laboratory or actual environmental samples were investigated, including the most recent findings published [44,76,92,93,94,95], *etc.* Detailed referenced work on this subject was shown in Kardinaal *et al.* [79] and Briand *et al.* [91]. Thus, the observed inter-site variations in intracellular MC concentrations in the Hartbeespoort Dam were not uncommon considering the size of the dam. Moreover, this study was arguably done during the summer season immediately before the onset of the colder season when *M. aeruginosa* undergoes overwintering [96,97], thus it is our assumption that we were able to capture typical trends in intracellular MC profiles from samples collected representing the dam situation during the summer season. Temporal distribution of *Microcystis* spp. responsible for MC production in the Hartbeespoort Dam has been demonstrated in the paper published by Van Ginkel *et al.* [47], however, quantitative evaluations of MC dominances were not shown. In her report Van Ginkel [24] reported that cyanobacterial cells increased in a pool with time from the beginning of the summer season and decreased at the onset of the colder season from March towards the winter months, a probable indication that *Microcystis* cells were overwintering. From the observations made during our study, the MC profiles are evenly distributed across the dam as they were shown to occur in all samples studied, implying that they are produced from one dominant and well-distributed species, *M. aeruginosa.* Our findings therefore corroborate those of previous studies which showed that *M. aeruginosa* is a dominant MC-producing species in the Hartbeespoort Dam [25,38,46,48,50,86,98].

**Table 1:** Summary of on-site water parameters observed from each sampling site between December 2010 and March 2011.

Month	Site	Temp(°C)	Surface water temperature (avg. °C)	Conductivity (µS/cm)	Dissolved oxygen (mg/L)	pH
	S1	24.6		578.9 ± 0.77	8.6 ± 0.35	8.4 ± 0.07
December	S2	25.2	24.8	586.3 ± 0.92	8.4 ± 0.28	8.2 ± 0.07
2010	S3	24.8		568.3 ± 1.55	7.7 ± 0.21	8.7 ± 0.07
	S4	24.7		561.9 ± 0.56	7.3 ± 0.21	8.5 ± 0.14
	S1	26.2		562.6 ± 2.47	7.3 ± 0.28	8.2± 0.07
January	S2	25.8	25.9	559.7 ± 1.13	7.8 ± 0.21	7.9 ± 0.07
2011	S3	25.4		558.9 ± 0.98	7.0 ± 0.14	8.4 ± 0.14
	S4	26.2		578.3 ± 1.2	7.3 ± 0.21	8.0 ± 0.07
	S1	25.2		555.3 ± 2.05	6.0 ± 0.35	7.8 ± 0.14
February	S2	25.7	25.3	549.3 ± 0.64	3.4 ± 0.35	7.4 ± 0.07
2011	S3	25.1		561.9 ± 1.69	3.8 ± 0.14	8.5 ± 0.14
	S4	25.3		554.9 ± 2.62	5.1 ± 0.28	8.3 ± 0.21
	S1	24.7		564.3 ± 0.64	6.2 ± 0.21	8.1 ± 0.14
March	S2	24.2	24.5	559.2 ± 1.48	3.3 ± 0.21	8.2 ± 0.14
2011	S3	24.9		560.5 ± 0.98	3.2 ± 0.14	8.7 ± 0.07
	S4	24.4		559.7 ± 1.13	3.5 ± 0.14	8.8 ± 0.21

**Table 2:** Site-specific monthly concentrations of each MC-congener, total MC concentrations (quantified MCs only) and content (%) of each congener (each concentration is a mean value of *n* = 3, ± STDev).

MC-Congener	Site S1				Site S2			
	Dec	Jan	Feb	Mar	Dec	Jan	Feb	Mar
**MC-RR**	21.96 ± 0.16	45.7 ± 2.9	50.46 ± 2.66	49.56 ± 1.62	88.56 ± 1.84	213.78 ± 17.76	268.16 ± 8.06	197.2 ± 2.87
**MC-YR**	7.24 ± 0.48	8.02 ± 0.4	14.12 ± 0.13	9.74 ± 0.58	15.78 ± 0.72	47.2 ± 1.58	71.22 ± 2.74	69.12 ± 1.4
**MC-LR**	6.46 ± 0.86	4.16 ± 0.54	17.26 ± 0.28	13.48 ± 0.52	57.58 ± 3.86	140.68 ± 2.88	128.66 ± 3.36	124.78 ± 3.02
**TOTAL MC (µg/g DW)**	35.66 ± 1.5	57.88 ± 3.84	81.84 ± 3.07	72.78 ± 2.72	161.92 ± 6.42	401.68 ± 22.42	468.04 ± 13.16	391.1 ± 7.29
**% MC-RR**	61.58	78.95	**61.65**	68.09	54.69	53.22	**57.29**	50.42
**% MC-LR**	18.11	7.18	21.08	18.52	35.56	35.02	27.49	31.9
**% MC-YR**	20.3	13.86	17.25	13.38	9.75	11.76	15.22	17.67
**MC-Congener**	**Site S3**				**Site S4**			
	**Dec**	**Jan**	**Feb**	**Mar**	**Dec**	**Jan**	**Feb**	**Mar**
**MC-RR**	5.94 ± 0.16	10.68 ± 0.34	16.78 ± 1.1	15.9 ± 0.94	13.78 ± 0.38	17.12 ± 0.2	15.98 ± 0.56	14.04 ± 0.64
**MC-YR**	0.22 ± 0.002	0.68 ± 0.04	3.34 ± 0.22	2.97 ± 0.28	0.14 ± 0.02	1.54 ± 0.02	1.72 ± 0.04	1.34 ± 0.06
**MC-LR**	5.24 ± 0.1	11.34 ± 0.18	9.12± 0.08	6.002 ± 0.2	3.78 ± 0.18	6.32 ± 0.08	5.82 ± 0.46	0.38 ± 0.04
**TOTAL MC (µg/g DW)**	11.4 ± 0.26	22.7 ± 0.56	29.24 ± 1.4	24.87 ± 1.42	17.7 ± 0.58	24.98 ± 0.015	23.52 ± 1.06	15.76 ± 0.74
**% MC-RR**	52.10	47.05 *	57.38	63.93	77.85	**68.53**	67.94	89.08
**% MC-LR**	45.96	49.95 *	31.19	24.13	21.56	25.3	24.74	2.41
**% MC-YR**	1.93	2.99	11.23	11.94	0.79	6.16	7.31	8.5

* Observed anomaly: MC-LR > MC-RR > MC-YR.

**Table 3:** Analysis of covariance (ANCOVA) statistical results indicating the effect of interaction between sites and MC congener on MC concentrations.

Entity	DF	Sum	Sq mean	Sq *F *value	Pr (>*F*)
MC	2	50.145	25.072	15.0204	1.806e−05 *
Site	3	261.023	87.008	52.1246	3.511e−13 *
MC:Site	6	58.292	9.715	5.8203	0.0002569 *
Residuals	36	60.092	1.669	-	-

* Significant difference at *P *< 0.05.

### 3.6. Health Implications of MC-RR Dominance and the Occurrence of (D-Asp^3^, Dha^7^)MC-RR Congener

From this study, the observed MC-RR concentrations in the dam were found to be between 5.94 µg/g and 268 µg/g DW (Table 2) among sites. This range of concentration is within and above the range detrimental to detoxifying organs found in fish, as described in the literature [99]. Based on mouse assay the toxicity level of MC-RR (LD_50_ = 200 µg/g to 800 µg/kg bw) is less than that of MC-LR (LD_50_ = 25 µg/g to 50 µg/kg bw) [100], however, Ito *et al.* [101] showed that both MC-RR and MC-LR equally inhibit the activities of PP1 and 2A enzymes. The inhibition of PP1 and PP2A activities results in metabolic dysfunction in animals and eventual death in case of higher dosages [31]. In addition, it has also been demonstrated that at elevated concentrations, MC-RR congener had inhibitory effects against antioxidant and detoxification activities of GST on fish gills [99,102] at a dose reaching 10 µg/L. MC-RR is the dominant congener in the Hartbeespoort Dam, toxicologically, however, the fact that its dominance would therefore pose the major health hazard may be ignored, since it is the uptake rate [90,103], synergistic effects of other compounds/or MCs [37,103] and the amount [104] of MCs ingested that are physiologically more important in terms of animal poisoning than toxicity values of any particular pure MC congener. For instance, bioaccumulation of MC-RR in fish and plant tissues resulting from its elevated extracellular concentration in water [104] is of importance, as toxicological effects pose health risks for the public consuming seafood products from this water as well as other uses of the water for domestic, recreational and agricultural activities. Although there is no guideline for MC-RR concentrations in recreational waters due to its low toxicity [100], the WHO has set a limit of 25 µg MC-LR eq/L for total MC concentration in recreational waters [105]. Therefore, from Table 2, it can be deduced that upon cell lysis in Hartbeespoort Dam by the end of the summer season, MC-RR would have contributed the highest proportion to the total MC concentration in recreational water, above the WHO threshold level for recreational waters. However, as described above, upon ingestion or dermatological contact, both MC-RR and MC-LR equally inhibit the activities of PP1 and 2A enzymes, thus elevated concentrations of MC-RR alone could potentially pose a health risk to the general consumers of products or services from the dam.

In addition, Blom and co-workers [104], showed that the toxicity activity of [D-Asp^3^, (E)-Dha^7^]MC-RR was higher than that of the MC-RR congener to algae grazers. Thus, although further investigation is still needed to fully characterise the structure of (D-Asp^3^, Dha^7^)MC-RR isolated from the algal extracts we studied, its occurrence in Hartbeespoort Dam is of particular interest to us with regard to what could be its synergistic effect (if any) on the toxicity of the dominant congener, MC-RR and the like.

## 4. Conclusions

The quantitative profiles, spatial distribution and the dominance of the MC-RR congener over MC-LR and MC-YR in Hartbeespoort Dam across all sites as well as the occurrence of MC-WR, MC-(H_4_)YR and (D-Asp^3^, Dha^7^)MC-RR in the summer season were reported and related to *M. aeruginosa *dominance. The information presented here about the identification of sites with higher MC concentrations, MC distribution and dominance in the dam is of particular importance to the Hartbeespoort Dam authorities, researchers and the general public for risk assessments and water safety purposes. The formulation of strategic interventions to minimise potential health risks to the public using such water is highly dependent on information of this kind, especially wherever high-risk sites are identified [69]. Thus, it is the authors’ view that information contained in this document has highlighted existing knowledge and has integrated the new knowledge with the existing knowledge to gain new insights into the state of safety of Hartbeespoort Dam water resources with respect to domestic, agricultural and recreational activities around and downstream of the dam.
